# Rational antibiotic use in China: lessons learnt through introducing surgeons to Australian guidelines

**DOI:** 10.1186/1743-8462-3-5

**Published:** 2006-05-30

**Authors:** Yan Zhang, Ken Harvey

**Affiliations:** 1 School of Public Health, La Trobe University, Bundoora, Vic, 3086, Australia

## Abstract

**Background:**

World-wide concern about increasing antibiotic resistance has focused attention on strategies to improve antibiotic use. This research adapted Australian best-practice guidelines on the prophylactic use of antibiotics in surgery to a Beijing teaching hospital and then used them as a quality assessment and improvement tool, supplemented by educational interventions. Qualitative data about factors influencing antibiotic use was also obtained.

**Methods:**

Australian and international guideline materials were amalgamated with the help of Chinese experts. Antibiotics prescribed for surgical prophylaxis in 60 consecutive patients undergoing clean or clean-contaminated surgery (120 total) were then compared with guideline recommendations in three phases; a pre-intervention period from June to August, 2002, an intervention period from June to August 2003 and post-intervention period from September to November 2003. During the intervention phase, feedback about prescriptions not in accord with the guideline was discussed with around 25 prescribers every two weeks. In addition, local factors influencing antibiotic use were explored with 13 junior surgeons and 8 high level informants.

**Results:**

While agreement was reached on the principles of antibiotic surgical prophylaxis there was no consensus on detail. Of 180 patients undergoing clean surgery throughout all phases of the study, antibiotic prophylaxis was administered to 78% compared to 98% of the 180 patients undergoing clean-contaminated surgery. Second and third generation cephalosporin antibiotics predominated in both low-risk clean and clean-contaminated operations. The timing of prophylaxis was correct in virtually all patients. The duration of prophylaxis was less than 24 hours in 96% of patients undergoing clean surgery compared to only 62% of patients undergoing clean-contaminated surgery. The intervention produced no improvement in the duration of prophylaxis nor the overuse and inappropriate choice of unnecessary broad-spectrum and expensive drugs. Interviews and focus groups revealed that an important explanation for the latter problem was Chinese government policy which expected hospitals to support themselves largely through the sale of drugs.

**Conclusion:**

Improving antibiotic use in China will require hospital funding reform, more authoritative best-practice guidelines, and hospital authorities embracing quality improvement.

## Background

Antibiotic guidelines and associated interventions have been demonstrated to be effective in improving antibiotic use. [1] Some countries, including Australia, have incorporated these principles into a national drug policy and provided government funding for a range of activities aimed at improving rational drug use. [2] Chinese hospitals and health organizations have become interested in such policies because of concern about inappropriate antibiotic prescribing [3,4] and a reported increase in the prevalence of antibiotic resistant organisms in Beijing, Shanghai and other big cities. [5-7] Antibiotic resistance has been described as a major threat to global public health by the World Health Organization because there are now few, and, in some cases, no antibiotics available to treat certain life threatening infections. [8]

Surgeons often use prophylactic antibiotics to prevent infection following operations and inappropriate antimicrobial prophylaxis is common. [9] This can be improved by guidelines and education. [10,11] In the year 2000, the pharmacy department of the Peking Union Medical College Hospital (PUMCH) translated the tenth edition of Australian Therapeutic Guidelines: Antibiotic [12] into Chinese and made it available for sale. Although these guidelines made recommendations for surgical prophylaxis, this material was simply an exact translation, it did not involve local medical or surgical experts and the drugs recommended were not always available locally. As a consequence, this initiative was considered to have made little impact.

This article outlines research aimed at more appropriately adapting Australian antibiotic guidelines for surgical prophylaxis to a Beijing hospital setting and then using them as a quality assessment and improvement tool, supplemented by educational interventions. Qualitative data about factors influencing antibiotic use was also obtained.

## Methods

### Study setting

The study site was a 120 bed general surgical unit within a 1200-bed general teaching hospital in Beijing. It was selected because the first author was a staff member but also because the hospital is a prestigious teaching institution, a model for other institutions and a source of influence on government policy. The attending surgeons and house residents had responsibility for antibiotic prescriptions while professors and senior doctors supervised the junior doctor's prescriptions.

### Guideline adaptation

Guideline adaptation commenced by amalgamating the Australian material mentioned above with Chinese [13] and other [14] material. The draft was then discussed with Professors of the Department of Surgery, hospital pharmacists, external microbiological experts and the hospital manager. There was agreement on the principles of timing (antibiotics to be administered within a two hour period before surgery commenced) and duration (no more than 24 hours) but disagreement on specific recommendations for particular procedures, including the recommendation that prophylactic antibiotics are not usually necessary for clean surgical operations. In addition, the hospital manager advised that the title should be changed to "Suggestion for Rational Antibiotic Use" because he felt that "guidelines" should be written by a national team of experts and have the approval of the Ministry of Health; a process that was not possible in the time available.

### Quantitative data collection

Quantitative data was collected from the surgical unit in three phases; pre-intervention period from June to August, 2002, the intervention period from June to August 2003 and the post-intervention period from September to November 2003. In each phase, 60 patients undergoing either clean or clean-contaminated operations were selected (a total of 120 in each phase). Thirty consecutive patients receiving operations for thyroid and breast disease respectively were used as representatives of clean operations (60 clean cases) while 30 consecutive patients receiving operations for gall bladder and gastric disease respectively represented clean-contaminated operations (60 clean-contaminated cases).

Detailed information was collected on patients (age, sex, surgical diagnosis, allergy, other diseases, operation time and complications), prescribers (age, sex, training period,) and the antibiotics used (generic names, doses, administrative method, timing, duration and any switch-over to another antibiotic). In each case, the antibiotics prescribed were compared with the agreed principles of surgical antibiotic prophylaxis and the results tabulated. The Chi Square test was used to analyses differences in categories of data such as the number of males and females and antibiotics prescribed in accord or not in accord with the agreed principles of timing and duration. The t-Test was used to evaluate the difference between average values for the three phases, such as age of patients, duration of operation, etc. SPSS version 11.0 was used for data analysis. A p value less than 0.05 was regarded as statistically significant.

### Educational intervention

Educational intervention was undertaken from June to August 2003. Feedback about prescriptions not in accord with the agreed principles was returned to around 25 prescribers every two weeks and a discussion on the principles of surgical antibiotic prophylaxis as they related to specific cases was offered to all staff interested. In addition, two tutorial/focus groups were organized for the education of 13 junior surgeons about surgical antibiotic prophylaxis. They were informed about the increased risks of antibiotic resistance, as well as the principles and the benefits of the rational choice of timing, duration, antibiotic selection. Data collection continued for three months after educational intervention (post-intervention study phase).

### Qualitative data collection

Qualitative methods were used to collect the opinions of high level informants and junior surgical staff about factors influencing antibiotic use. Structured interviews were conducted with the Hospital Manager, Hospital Director (also a council member of the State Drug Committee), Vice Director of Pharmacy, Dean of the Surgical Department, an official from the Ministry of Health, the Manager of the local State Food and Drug Administration and another council member of the State Drug Committee (also Manager of another hospital) (a total of eight key informants). Two focus groups were also held with a combined total of 13 junior hospital surgical staff (the majority of the junior surgical staff).

## Results

### Patients and surgeons

No statistically significant difference was found between the patients in the pre-intervention, intervention and post-intervention phases of the study with regard to patients' sex, average age, average operation duration, rate of associated disease (diabetes) and postoperative complications. Female patients predominated (70%) because patients with breast disease were specifically selected. There were a similar number of general surgeons involved in the three study phases (about 26) and no significant difference with regard to their age and the training period. The prescribing surgeons who were exposed to the intervention were the same staff who were followed up in post intervention phase.

### Timing and duration of prophylactic antibiotics

There was no significant difference between patients in the pre-intervention, intervention and post-intervention phases of the study regarding the timing or duration of antibiotics used. For all groups, almost all antibiotics were given by the intravenous route (99%) and there were a similar number of antibiotics used per patient (average of 1.23). Almost all patients (99%) had antibiotics administered within a two hour period prior to the commencement of surgery. Almost two thirds of the patients receiving antibiotics were given a single dose only; the median duration of administration was 1 day, the mean 1.7 days and the range 1 to 19 days.

Because there was no statistically significant difference between results in the control, intervention and post-intervention groups, these were combined and then separated into patients undergoing clean and clean-contaminated surgery. There was a high rate (78%) of antibiotic prophylaxis for patients undergoing clean surgery and an even higher rate (98%) for patients undergoing clean-contaminated surgery. There were no post-operative infective complications recorded in any of the clean and clean-contaminated operations studied. Many more patients undergoing clean-contaminated surgery received antibiotic prophylaxis for greater than 1 day (38%) compared to those undergoing clean surgery (4%).

### Choice of prophylactic antibiotics

In order to fairly analyze the choice of antibiotics in clean and clean contaminated operations we excluded cases of malignant tumour, malnutrition, diabetes, anaemia, cirrhosis and patients over 70 years where the surgeon may have been concerned about an increased risk of post-operative infection. In 94 clean operations, second and third generation cephalosporin antibiotics or the fluoroquinolone levofloxacin accounted for 96% of the antibiotics used. In 82 clean-contaminated operations, second and third generation cephalosporin antibiotics or levofloxacin were used in all cases; in a third of patients these drugs were also combined with metronidazole.

### Qualitative results

Interviews and focus groups provided additional information about factors influencing antibiotic use. The translated Australian antibiotic guideline book was not regarded as practical because its recommendations were for generic drugs whereas the hospital drug list (and all prescribing) used brand names. In addition, the book had to be purchased whereas information from pharmaceutical companies was free. Table 1 summarises the major factors that respondents felt influenced antibiotic use.

**Table 1:** Factors influencing antibiotic use in China

Factor	Influence
Government policy	By poorly remunerating doctors and expecting hospitals to support themselves largely through the sale of drugs, government policy encouraged over-prescribing of expensive drugs and discouraged quality assessment and improvement exercises.
Pharmaceutical industry	By spending a large amount of money on drug advertising, gifts and financial "kick-backs" to doctors who prescribed their drugs drug companies encouraged excessive prescribing ("kick-backs" were particularly attractive given the low salary of hospital doctors).
Hospital Drug and Therapeutics Committees	Generally regarded as ineffective; in particular they provided no monitoring of prescriptions and little independent education to medical staff.
Surgeons attitude and knowledge	By being less interested in drugs than physicians ("operations were more important") misunderstandings were perpetuated such as, "new antibiotics are stronger"; "new drugs kill most germs"; "the bigger the operation, the greater the need for newer and stronger antibiotics".
Deteriorating relationship between doctors and patients	This led to doctors protecting themselves from being sued by prescribing unnecessary &/or expensive drugs; this practice was often acerbated by media reports of patients physically assaulting the medical staff &/or extorting money from hospitals when treatment failed.

Suggestions for improving antibiotic use included improving health financing systems (including patient compensation schemes to limit doctors being sued), attracting support and permission from the authorized organizations such as the Ministry of Health for developing authoritative best-practice guidelines and educational interventions, and improving therapeutic education and monitoring within hospitals.

## Discussion

Burke [9] noted that inappropriate antimicrobial prophylaxis for surgical patients was common. Important errors included delays in administering antibiotics, excessive duration and the use of inappropriate agents. In this study, the timing of antibiotics used for surgical prophylaxis was regarded as satisfactory. In all groups studied, 99% of prophylactic antibiotics were prescribed within a two-hour period immediately before the operation (usually administered intravenously at anaesthetic induction). With respect to duration, almost two thirds of the patients received a single dose, while a little over three quarters had antimicrobials administered for one day or less. However, only 62% of patients undergoing clean-contaminated surgery received antimicrobials for one day or less compared to 96% undergoing clean surgery. The former is not in accord with established guidelines. In addition, prophylactic antibiotics were administered to 78% of low-risk clean surgical operations and 98% of clean-contaminated operations; a very high use compared with other studies. [15,16]

In most patients undergoing clean surgery the risk of infection is very low and the guidelines we consulted did not routinely recommend prophylactic antibiotics unless the patient was immunosuppressed or otherwise predisposed to infection. In the latter situation, the most likely contaminating micro organisms will be skin flora such as Staphylococcus aureus and possibly Streptococcus pyogenes. The prophylactic antibiotic of choice will be a narrow-spectrum beta-lactamase stable penicillin or a first-generation cephalosporin such as cefazolin (often favoured because of its longer half-life). [17] However, we found extensive use of more expensive, second and third generation cephalosporins or fluoroquinolones whose activity against Gram-negative organisms was unnecessary.

The clean-contaminated cases analysed comprised gastric or gall bladder surgery with complications, such as obstruction and diabetes, excluded. The risk of infection, although higher than in clean surgery, is still low and the likely contaminating organisms are aerobic Gram-negative organisms (in addition to skin flora). Anaerobic infection in this situation is most unlikely. However, we found that third-generation cephalosporin antibiotics and fluoroquinolones, often in association with metronidazole, were commonly used in this situation rather than second-generation cephalosporin antibiotics recommended by many authorities. [13,18]

The unnecessary use of extended spectrum antibiotics for surgical prophylaxis encourages the selection of resistant micro organisms, causes more patient adverse events and wastes money. In particular, the use of third-generation cephalosporins is a risk factor for infection with methicillin-resistant Staphylococcus aureus, Clostridium difficile, enterococci and resistant gram-negative bacilli. [19] These antibiotics, along with vancomycin and metronidazole, have been significantly associated with vancomycin-resistant enterococci (VRE) [20].

The educational intervention produced no statistically significant difference in either the duration or the timing of antibiotics used in the pre-intervention, intervention and post-intervention phases studied. These principles were already being well observed (especially timing) and thus the study lacked statistical power to detect a small added improvement. However, other factors were also likely to have been involved, especially in failing to shorten the duration of prophylaxis. The Chinese researcher was relatively junior and lacked influence in a hierarchal institution. Agreement could not be reached on specific recommendations for particular procedures, including the recommendation that prophylactic antibiotics are not usually necessary for clean surgical operations. In addition, the hospital manager felt that "guidelines" should be written by a national team of experts and have the approval of the Ministry of Health; he advised that the title should be changed to "Suggestion for Rational Antibiotic Use".

Furthermore, although the educational intervention stressed the principles of appropriate antibiotic selection, qualitative research into the barriers to rational prescribing revealed other powerful influences that encouraged the use of unnecessarily expensive broad-spectrum antibiotics; in particular the perverse effect of government policy which expected hospitals to support themselves largely through the sale of drugs. This meant that any attempt to produce a more discriminating use of antibiotics would have an adverse effect on hospital finances. In addition, prescribers had particular personal and social constraints such as insufficient knowledge, low salary, concerns about litigation by patients and excessive reliance on the pharmaceutical industry (including susceptibility to "kick-backs"), all of which shaped their attitude towards rational antibiotic use.

## Conclusion and recommendations

While this research failed in its prime aim of improving the use of prophylactic antibiotics by surgeons in a Chinese hospital it succeeded in documenting particular problems of inappropriate antibiotic use. In addition, the research suggested that the key determinant of this behaviour lies outside any particular prescriber or hospital and relates to government policy concerning hospital financing. A pragmatic solution to the latter problem might be to increase patient fees for medical services while lowering the profit hospitals obtain by selling drugs. A national campaign to inform and educate the Chinese public on rational and economic use of medicine is also required. In the longer term, there is a need to explore national public insurance schemes as a better way of more equitably distributing health care costs. This would require bringing together important stake-holders such as consumers (through consumer organizations/civil society), health policy makers, prescribers and the media to achieve consensus and lobby the government to include national health insurance as a policy priority. A draft national charter of patients rights and responsibilities would also be a useful campaigning tool.

Finally, there is also the need for a process, both nationally and within hospitals, to facilitate clinicians reaching agreement about evidence-based guideline recommendations, fund education campaigns to explain why guidelines are being introduced and conduct regular drug utilization studies to track the quality of prescribing.

## Postscript

In 2004, the Chinese Ministry of Health issued "Principles of Clinical Antimicrobial Use Guidelines". This manual aimed to standardize doctors' use of antibiotics and improve treatment of bacterial infections in Chinese hospitals. In November 2005, a National Workshop on Rational Use of Antibiotics was held at the Beijing Children's Hospital. The participants made a number of recommendations very similar to those suggested in this paper.

## Competing interests

The author(s) declare that they have no competing interests.

## Authors' contributions

YZ reviewed the literature and carried out the research in the field, KH assisted with study design, supervision and writing.

## References

[B1] Harvey K, Dartnell J, Hemming M (2003). Improving antibiotic use: 25 years of antibiotic guidelines and related initiatives.. Commun Dis Intell.

[B2] Australia C, Commonwealth of Australia (2002). The National Strategy for Quality Use of Medicines.

[B3] Li H, Li X, Zeng X (1995). A study on antibiotic abuse in 750 children with acute respiratory infection in Tongxian County of Beijing. Zhonghua Yu Fang Yi Xue Za Zhi.

[B4] Yang YH, Fu SG, Peng H, Shen AD, Yue SJ, Go YF, Yuan L, Jiang ZF (1993). Abuse of antibiotics in China and its potential interference in determining the etiology of pediatric bacterial diseases. Pediatr Infect Dis J.

[B5] Li J, Weinstein AJ, Yang M (2001). Surveillance of bacterial resistance in China (1998-1999). Zhonghua Yi Xue Za Zhi.

[B6] Liu GJ, Xu SZ, Ma Y, Li JY (2003). Analysis of antimicrobial resistance of clinical isolates of enterococci from Beijing and other areas in China. Zhonghua Yi Xue Za Zhi.

[B7] Wang F, Zhu DM, Hu FP, Zhang YY (2001). Surveillance of bacterial resistance among isolates in Shanghai in 1999. J Infect Chemother.

[B8] World Health Organisation (2000). Antimicrobial resistance: the facts. WHO: Essential Drugs Monitor.

[B9] Burke JP (2001). Maximizing Appropriate Antibiotic Prophylaxis for Surgical Patients: An Update from LDS Hospital, Salt Lake City. Clinical Infectious Diseases.

[B10] Lallemand S, Albin C, Huc B, Picard A, Roux C, Tuefferd N, Talon D (2002). Evaluation of practices in surgical antimicrobial prophylaxis in the Franche-Comte before and after implementation of an information program. Ann Fr Anesth Reanim.

[B11] Landgre FT, Harvey KJ, Mashford ML, et al (1988). Changing antibiotic prescribing by educational marketing. Med J Aust.

[B12] Antibiotic Writing Group (1998). Therapeutic guidelines: Antibiotic..

[B13] Li ZL (2001). Principles and difference between prophylactic and therapeutic antibiotic use in surgery. Zhongguo Shi Yong Wai Ke Za Zhi.

[B14] Bohnen JMA, Solomkin JS, Dellinger EP, Bjornson HS, Page CP (1992). Guidelines for clinical care: anti-infective agents for intra-abdominal infection. Arch Surg.

[B15] Vaisbrud V, Raveh D, Schlesinger Y, A.M. Y (1999). Surveillance of antimicrobial prophylaxis for surgical procedure. Infect Control Hosp Epidemiol.

[B16] Bellomo R, Bersten AD, Boots RJ, Bristow PJ, Dobb GJ, Finfer SR, McArthur CJ, Richards B, Skowronski GA (1998). The use of antimicrobials in ten Australian and New Zealand intensive care units. The Australian and New Zealand Intensive Care Multicentre Studies Group Investigators..

[B17] Swedish-Norwegian Consensus Group (1998). Antibiotic prophylaxis in surgery: summary of a Swedish-Norwegian Consensus Conference. Scand J Infect Dis.

[B18] Paterson DL, Playford EG (1998). Should third-generation cephalosporins be the empirical treatment of choice for severe community-acquired pneumonia in adults?. Med J Aust.

[B19] Dahms RA, Johnson EM, Statz CL, et al (1998). Third-generation cephalosporins and vancomycin as risk factors for postoperative vancomycin-resistant enterococcus infection. Arch Surg.

